# In search of a frame: challenges and opportunities for sampling immigrant minorities

**DOI:** 10.1186/s40878-018-0103-5

**Published:** 2018-11-26

**Authors:** Romana Careja, Hans-Jürgen Andreß

**Affiliations:** 1 Syddansk Universitet, Odense, Denmark; 2 Universität zu Köln, Cologne, Germany

**Keywords:** Sampling, Immigrants, Population registers, Western Europe

## Abstract

When it comes to evaluating immigrants’ integration, survey data are particularly important. However, the endeavor of surveying immigrant minorities is challenging. This special issue focuses on the possibility of obtaining high-quality cross-country comparable samples of immigrant minorities. As the golden standard for drawing random representative samples are the population lists, the contributions of the special issue have directed their attention to this sampling frame. In the three country-based articles, the authors are discussing the legal, administrative and scholarly challenges and opportunities for using population registers as sampling frames for immigrant minorities. The fourth article summarize their findings and concludes that identical register-based sampling design is difficult to implement in a cross-country design, due to the significant differences in the quality of population registers and their accessibility to researchers. The authors suggest that by sampling immigrant minorities in cities, researchers can better implement sampling strategies which result in comparable samples.

With the recent increased influx of immigrants, the policy and academic worlds faced a re-newed need of information about immigrant minorities^1^ entering and/or already residing on the territories of the European countries. The need for comprehensive and reliable data has been widely acknowledged by the European Union and international statistical bodies such as the United Nations, the OECD, and the World Bank. The EU regulation (EC) 862/2007 on community statistics on migration and international protection, the Declaration of Zaragoza (2010) stressing the need to have common indicators, or the Task Force on Improving Migration and Migrant Data Using Surveys and Other Data, also known as the ‘Suitland Working Group’, are examples of more concrete steps in the quest for better data.

Responding to these initiatives, many European countries have increased their efforts to improve their national statistical infrastructure to account for immigrant minorities. However, a major issue remains the question of how these new populations integrate into the destination countries and how the integration process can be managed by integration policies. The answer to these questions, not only requires *more* data, but also *more complex* data on immigrant minorities.

In the last 20 years the regulatory context regarding entry and stay in the Western European countries has changed dramatically, compared to most of the second half of the twentieth century. Moreover, recent research has shown that across European countries there are significant differences in the way immigrant minorities’ access to economic, social and political arenas is regulated. For a better understanding of the effects these differences make on their integration, data on the living conditions and attitudes of immigrant minorities in different countries are necessary. Which means that, in order to allow valid cross-country *comparisons*, these data have to be collected in an identical (comparable) way in all countries of interest.

It is fair to say that, currently, comparative individual-level data from immigrant minorities remain rare. Cross-national survey projects such as the European Social Survey (ESS), the International Social Survey Programme (ISSP), the Eurobarometer surveys, or the European Value Study (EVS) capture only a small number of migrants and need to be pooled over time to achieve sufficient sample sizes. The only exception among these cross-national surveys is the European Labour Force Survey (LFS), which due to its huge sample size also includes many migrants, but only monitors the labor market situation of individuals. Country-level surveys such as the German Socio-Economic Panel (SOEP) or the Longitudinal Internet Studies for the Social Sciences (LISS) in the Netherlands over-sample immigrant minorities, but are not comparable across countries as the sampling methodology and the questionnaires differ. Sometimes, surveys focus only on certain regions within one country, as does the Osservatorio Regionale per L’integrazione e la Multietnicità (2001–2016) which is undertaken in Lombardy, an Italian region. Several specialized survey programs on immigrant minorities have been started only recently. Some collect data at national level, such as the IAB-SOEP Migration Sample in Germany, while others collect data across borders (see for example the NORFACE Research Programme on Migration or the EU research programs (FP7, HORISON 2020) with several projects interested in migration).

In parallel to these data collection endeavors, the methodological literature reveals an increasing interest in exploring, understanding and developing tools to overcome the challenges of data collection on and from immigrant minorities. In the aftermath of efforts spearheaded by the EU to develop a common framework for assessing immigrants’ numbers and integration (see Kraler, Reichel, & Entzinger, 2015 for an overview), several volumes have taken stock of the statistical infrastructure and the conceptual frameworks that EU member states employ to monitor the immigrant minorities resident on their territories. For example, the volume edited by Fassmann, Reeger, and Sievers (2009) focuses on flows and stocks, while the volume edited by Bijl and Verweij (2012) focuses on immigrants’ integration. The country-studies in these volumes illustrate how some countries use surveys to monitor immigrants’ integration and cross-border mobility, while others rely mostly on administrative data. Both volumes document the significant differences across the EU member states, not least due to the different experiences with immigration and the preparedness of their administrative systems to include the new residents. In addition, there are research projects involved in evaluating the available data and the data-sources: We highlight here two large such projects, PROMINSTAT (with a focus on discrimination and integration, see Kraler & Reichel, 2010) and CLANDESTINO (with a focus on illegal and undocumented immigrants, see Jandl, Vogl, & Kraler, 2008).

When it comes to evaluating immigrants’ integration, survey data are particularly important. The reason is that in addition to objective information (such as labor market position and history, or social networks), they offer a wealth of subjective information which provide deeper insights into the connection between immigrants and the countries where they reside. However, collecting data by surveying immigrants is no easy feat, as challenges occur at each step. To the best of our knowledge, in addition to several working papers or articles published in separated outlets (Deding, Fridberg, & Jakobsen, 2008; Reher & Requena, 2009; Ahlmark et al., 2015; Platt, Luthra, & Frere-Smith, 2015), there are four volumes published in the 2000s addressing different aspects of collecting data on immigrant minorities via surveys (Bonifazi, Okolski, Schoorl, & Simon, 2008; Vargas-Silva, 2012; Font & Mendez, 2013). These volumes capture a longstanding preoccupation with the issue of producing high quality surveys, while mitigating the problems arising from the limited availability or outright unavailability of sampling frames, or from the changing nature of the target group. However, a gap in this literature can be identified: without diminishing in any way the contribution of these studies to understanding the problems and the advances in this field of research, we believe that what is missing is a systematic cross-country analysis of a particular core-issue in survey research with a focus on immigrant surveys. Following an extended discussion with experts in several countries, we have identified this *core issue as the drawing of high-quality cross-country comparable samples of immigrant minorities*.

This special issue addresses this gap by:

- Explicit focus on sampling immigrant minorities: We recognize that, as illustrated by previous publications, the study of immigrants lends itself to a large variety of approaches and methods. However, this special issue focuses explicitly on surveys, and within this area, on the matter of sampling, because we believe that it is a core issue which decisively determines the quality of the data collected. This focus on surveys of immigrant population implies that the contributions do not include discussions of general population surveys that capture immigrants in their sample. Immigrant surveys face specific sampling problems, which need a discussion separated from sampling for general population surveys.
- Systematic country-by-country analysis of the legal and administrative context for surveying immigrants: The special issue includes three articles which elaborate on existing sampling frames (or absence thereof) in two countries from Northern Europe (Sweden, Denmark), two from Continental Europe (Germany, The Netherlands), and two from Southern Europe (Spain, Italy). The contributors were asked to describe the legal and administrative conditions for data access, and the type of data available. In so doing, the contributions move beyond the data availability issues and discuss whether and how the statistical information available in each country can be used to develop usable sampling frames.
- Illustrations of how the sampling frames are used or built in the selected countries: The articles include examples of immigrant surveys conducted in the selected countries to illustrate different possibilities to develop such surveys given the existing sampling frames and by discussing possible trade-offs between sampling strategies.
- Systematic approach: By asking our contributors to follow the same structure across the chapters, and address similar questions, the special issue results in a systematic examination of the possibilities to survey immigrant minorities in the selected EU countries. Ultimately, this approach enables us to answer questions about the feasibility of cross-country comparative surveys.

The article by *Kurt Salentin* and *Hans Schmeets* discusses the limitations of harmonized sampling designs for survey research on immigrants in Germany and the Netherlands (Salentin & Schmeets, 2017). Although both countries use largely similar definitions and categories of immigrant minorities, the two countries differ in data accessibility. In the Netherlands a country-level sample can be drawn from a national population register, but only by Statistics Netherlands. However, this is impossible in Germany due to the decentralized setup of the population register and legal restrictions on merging existing databases. This leads the authors to propose that a two-stage strategy, in which first municipalities and second individuals are sampled, seems most likely appropriate for these two countries. However, they note that rules and regulations might severely constrain implementation. The authors conclude their analysis by pointing out that even if a common sampling frame can be developed given the existence of common definitions of the population of interest, achieving a harmonized data collection can remain a major challenge due to legal and administrative restrictions.

The article by *Inmaculada Serrano Sanguilinda*, *Elisa Barbiano di Belgiojoso*, *Amparo González Ferrer*, *Stefania Maria Lorenza Rimoldi* and *Gian Carlo Blangiardo* brings forth the challenges raised in southern European countries, Italy and Spain, by increased recent immigration, including high numbers of irregular immigrants (Serrano Sanguilinda, Barbiano di Belgiojoso, González Ferrer, Rimoldi, & Blangiardo, 2017). Although both countries have some form of population registries, they answered the challenges differently: While Spain introduced significant improvements to its register Padrón, resulting in increased coverage and accuracy, Italy did not do so. Consequently, Padrón is likely to provide a reasonably good sampling frame, while Italy’s Anagrafe not, forcing the researchers to resort to non-random sampling methods. The article discusses the methodological implications of these differences and evaluates different methodological solutions based on both random and non-random sampling methods in both countries.

The article by *Romana Careja* and *Pieter Bevelander* uses the cases of Denmark and Sweden, where centralized registers produce detailed records about all residents’ lives (Careja & Bevelander, 2018). The study finds that Danish and Swedish registers provide systematic objective data which are fully available to researchers, but are unable to provide data to answer questions regarding the deeper aspects of integration processes, such as opinions and preferences of immigrants. Although registers do not collect subjective data, they can be used as sampling frames for obtaining random immigrant samples. The authors however identify and discuss several traits of the registers likely to affect the representativeness of the desired immigrant samples.

On the basis of the information in these three articles, the conclusion of the special issue evaluates the feasibility of cross-country sampling strategies which yield high quality samples of immigrant minorities. The article by *Hans-Jürgen Andreß* and *Romana Careja* focuses on probability samples and the use of population registers, as these are the standard for quality samples, while other sampling strategies are only briefly touched upon (Andreß & Careja, 2018). The analysis shows that even with only six European countries an identical register-based sampling design is difficult. The authors propose that, by focusing on sampling immigrant minorities in cities, researchers can better implement sampling strategies which result in comparable samples.

This Special Issue was launched as a forum for discussing the possibilities to draw of high-quality cross-country comparable samples of immigrant minorities. Previous research has shown that more often than not, practical constraints have pushed researchers to trade-off the requirement for sample randomness over study feasibility. Good practices such as keeping detailed records of sampling decisions help in minimizing the risk of bias (see for example discussions in various chapters in Font and Mendez (2013) and Bonifazi et al. (2008)), but may be of little help to assuage critical voices concerned with the quality of the resulting samples.

As the gold standard in social sciences is sampling based on population lists (registers), the Special Issue focused on the possibility of using them to draw representative samples of immigrant minorities, samples which would satisfy the randomness criterion and would allow cross-country comparative studies. In this respect, the main finding seems to be a negative one: an identical register-based sampling design is difficult to implement in several countries, mainly due to the fact that countries differ significantly in the quality of their population registers, as well in the access they allow to these population lists. The Special Issue thus corroborates the findings of previous studies, which illustrate the various challenges survey researchers encounter when dealing with immigrant minorities. The concluding article of the Special Issue encourages researchers – to the extent that it is possible and fitting with the intended research project/question – to rescale their comparative projects in such a way as to take advantage of sampling frames which allow extracting samples which fulfill the criteria of randomness and comparability – for example by sampling immigrant minorities in urban areas.

## Footnotes

1 The term “immigrant minorities” designates ethnic minority groups who originate from migratory processes. The term has been around for long time (see for example Schmitter, 1983; Geddes, 1995; Sniderman, Hagendoorn, & Prior, 2004; Dowley & Silver, 2011; Hainmueller & Hangartner, 2012; De Vroome, Hooghe, & Marien, 2013; Schaeffer, Höhne, & Teney, 2016) and includes both recently arrived immigrants and individuals settled in the territory of the host countries, as well as their offspring. The term “immigrant minorities” also allows a distinction from “national minorities”, who are ethnic groups who have lived in these European countries for centuries.
